# Altered Functional Connectivity and Cognition Persists 4 Years After a Transient Ischemic Attack or Minor Stroke

**DOI:** 10.3389/fneur.2021.612177

**Published:** 2021-06-07

**Authors:** Korinne Nicolas, Peter Goodin, Milanka M. Visser, Patricia T. Michie, Andrew Bivard, Christopher Levi, Mark W. Parsons, Frini Karayanidis

**Affiliations:** ^1^Functional Neuroimaging Laboratory, School of Psychology, University of Newcastle, Callaghan, NSW, Australia; ^2^Hunter Medical Research Institute, Newcastle, NSW, Australia; ^3^Priority Research Centre for Stroke and Brain Injury, The University of Newcastle, Callaghan, NSW, Australia; ^4^Melbourne Brain Centre, Royal Melbourne Hospital, University of Melbourne, Parkville, VIC, Australia; ^5^Sydney Partnership for Health, Education, Research and Enterprise, Sydney, NSW, Australia

**Keywords:** transient ischaemic attack, minor stroke, cognitive impairment, functional connectivity, executive function

## Abstract

**Background and Purpose:** Altered executive functions and resting-state functional connectivity (rsFC) are common following a minor stroke or transient ischemic attack (TIA). However, the long-term persistence of these abnormalities is not well-studied. We investigated whether there were cognitive and rsFC differences between (a) controls and minor cerebrovascular event (CVE) patients and (b) between CVE patients with and without an imaging confirmed infarct (i.e., minor stroke and TIA, respectively) at an average of 3.8 years following their event.

**Methods:** Structural and resting-state imaging and cognitive assessments including the Montreal Cognitive Assessment, the Trail Making Task and the National Institute of Health (NIH) Cognition Toolbox were conducted on 42 patients (minor stroke = 17, TIA = 25) and 20 healthy controls (total *N* = 62).

**Results:** Controls performed better than patients on two measures of executive functioning (both *p* < 0.046) and had reduced rsFC between the frontoparietal and default mode networks (FPN and DMN, respectively; *p* = 0.035). No cognitive differences were found between minor stroke and TIA patients, however, rsFC differences were found within the FPN and the DMN (both *p* < 0.013). Specifically, increased connectivity within the FPN was associated with faster performance in the minor stroke group but not the TIA group (*p* = 0.047).

**Conclusions:** These findings suggest that transient or relatively minor cerebrovascular events are associated with persistent disruption of functional connectivity of neural networks and cognitive performance. These findings suggest a need for novel interventions beyond secondary prevention to reduce the risk of persistent cognitive deficits.

## Introduction

Minor stroke and transient ischemic attack (TIA) are cerebrovascular events (CVEs) characterized by a brief mild ischaemic episode with transient clinical and neurological symptoms. Disturbances in executive functions are well recognized following CVEs and include impairments to attention, memory, planning and cognitive flexibility (1, 2). Executive dysfunction can reduce a patient's ability to undertake the activities of daily living which can reduce one's quality of life and the likelihood of returning to premorbid levels of functioning (3). Studies show substantial variability in the reported prevalence of moderate (29–68%) or severe (8–22%) executive dysfunction following a minor CVE. These discrepancies may arise from a range of methodological differences, including inconsistency in neurological event definition. The classical, clinical definition differentiates between minor stroke and TIA based on symptom duration, however, the more recent definition, based on diffusion-weighted magnetic resonance imaging (MRI) (4) differentiates minor stroke from TIA using confirmation of acute tissue infarction (5). Many studies, however, continue to use the classical definition (6–8) due to convenience or lack of access to advanced brain imaging which can be problematic, as studies comparing patients using the two definitions show that up to 30% of conventionally-defined TIA patients display cerebral infarction on MRI (9, 10). Moreover, many studies do not control for variables that independently affect cognition [e.g., age, presence of vascular risk factors, premorbid functioning (10)] or they pool minor stroke and TIA patients together, making it unclear whether there are structural or functional differences between minor CVE patients with persistent ischaemic damage (i.e., minor stroke) and without damage (i.e., TIA). Finally, most studies assess patients in the acute or subacute stages after a minor CVE when variability is likely to be maximal (10), and only a few have investigated the long-term impacts of such events (11, 12).

By using resting-state functional connectivity (rsFC) analysis of functional MRI (fMRI), previous work (13–15) have found that compared to healthy controls, TIA patients typically display patterns of rsFC indicative of reduced executive functions, including decreased rsFC in prefrontal neural regions and increased connectivity between task-positive and task-negative networks [e.g., the frontoparietal network; FPN and the default mode network; DMN, respectively (16, 17)]. Moreover, Guo et al. (18) found these functional connectivity differences in TIA patients were associated with reduced cognitive ability on a broad screening test. Moreover, Bivard et al. (19) reported that patients with a TIA (defined as a baseline hemispheric perfusion deficit without evidence of an ischemic lesion on 24 h MRI) displayed significantly greater gray matter atrophy and poorer performance on the Montreal Cognitive Assessment 90 days following their event, compared to healthy controls. Thus, there is strong evidence for widespread disruption of rsFC and cognition in acute and subacute periods following a TIA. However, there is limited evidence regarding the longer-term cognitive and functional connectivity outcomes. Further, there is little evidence of the functional connectivity outcomes of a minor stroke.

This study aims to assess patients with a clinically and radiologically diagnosed minor stroke or TIA on average 4 years post-event to identify whether they have persistent neurological impairments on resting-state MRI and general cognitive assessments. We hypothesize that, compared to healthy controls, CVE patients will show (a) reduced executive functions and (b) reduced internetwork (between the FPN and DMN) and increased intranetwork (within the FPN and the DMN) rsFC. Further, as a CVE with persistent tissue damage may have a greater impact on brain function, we hypothesize that patients with MRI-defined infarction (i.e., minor stroke patients) will display lower cognitive ability, reduced internetwork rsFC and increased intranetwork rsFC compared to patients with no evidence of infarction (i.e., TIA patients).

Finally, we will test the above hypotheses after correcting for the effects of vascular risk factors and age which both increase the risk of developing a CVE and are independently associated with executive functions (20–22). These parameters will assist in determining whether cognitive or functional connectivity differences, if present, are associated with the minor CVE or a chronic cerebrovascular disease process which may be associated with aging.

## Methods

### Participants

Minor CVE patients were recruited from two sources. Fifteen patients were recruited from a general neurology clinic in the Hunter region based on a clinical diagnosis of a possible minor stroke or TIA within the last 3 years. Eighty-two patients were recruited from the International Systems of Care and Patient Outcomes in Minor Stroke and TIA (INSIST) study, a longitudinal, community-based inception cohort study that recruited patients with a possible stroke or TIA diagnosis from general practices, emergency departments, and acute cerebrovascular units in the Hunter and Manning Valley regions of New South Wales, Australia (23). In the INSIST study, event adjudication was completed by an expert panel using a standardized clinical definition along with clinical notes and medical history (see Table 1). The INSIST-COG study was approved by Hunter New England Health and the University of Newcastle Human Research Ethics Committees (12/04/18/4.02; H-2012-0154). All patients provided written informed consent.

**Table 1:** Criteria for adjudication of minor stroke and TIA event types in the INSIST and INSIST-COG cohorts.

**Event type**	**INSIST criteria**
Minor stroke	A stroke with a National Institutes of Health Stroke Scale score of ≤ 4 lasting more than 24 h
Transient ischemic attack	Rapidly developing clinical signs of focal disturbance of cerebral function lasting <24 h with no apparent non-vascular cause.
	**INSIST-COG criteria**
Minor stroke	Clinically diagnosed minor cerebrovascular event patients from the INSIST study or practicing neurologist with evidence of infarction including signs such as localized atrophy and signal hypoattenuation
Transient ischemic attack	Clinically diagnosed minor cerebrovascular event patients from the INSIST study or practicing neurologist with no evidence of infarction.

Healthy controls were recruited from the Hunter Medical Research Institute (HMRI) volunteer registry, community groups and social media. Controls were selected to have no history of a neurological or vascular event, radiation therapy within the last 5 years, any neurodegenerative disorders and to have fewer than three vascular risk factors. All participants gave written informed consent and the study was approved by the Hunter New England Human Research Ethics Committee (12/04/18/4.02).

All INSIST-COG participants completed an MRI scan (see below). Minor CVE patients were reclassified based on the presence of an infarct on the diffusion weighted MRI evidence by a radiologist blinded to prior event history as either having a minor stroke or a TIA. Specifically, patients were classified as having had a minor stroke if there was evidence of infarction including signs such as localized atrophy and signal hypoattenuation. If there was no evidence of infarction, they were classified as having had a TIA (see Table 1). Thus, patients in the current study were initially clinically adjudicated as having had a minor CVE (INSIST or clinical notes), and later the DWI was used to confirm the absence or presence of an infarct and classify as TIA or minor stroke, respectively (see Table 2).

**Table 2:** Reclassification of clinically diagnosed minor cerebrovascular event patients following diffusion weighted MRI ~4 years after their event.

**Clinical event diagnosis **~**4 years prior from INSIST study and neurologist clinic**	**Evidence of MRI infarct at present date**	**No Evidence of MRI infarct at present date**
Minor stroke *N* = 13	10	3
TIA *N* = 29	7	22
INSIST-COG reclassification	Total = 17 minor stroke patients	Total = 25 TIA patients

### Procedure

Participants attended two testing sessions [median 13 days apart: interquartile range (IQR) 5 and 32]. Demographic information, health and lifestyle history, and brief cognitive and affective questionnaires were obtained in the telephone interview (session 1). Cognitive testing and an MRI were completed during session 2.

### Imaging

Scans were undertaken on a 3T Prisma (Siemens Healthineers, Erlangen, Germany) MRI scanner with a 64-channel head and neck coil. For each subject, a high-resolution T1-weighted anatomical image was acquired in the sagittal plane (TR = 2.3 s, TE = 2.96 ms, inversion time = 900 ms, flip angle = 9°, FOV = 256 mm, voxel size = 1.0 × 1.0 × 1.0 mm, total acquisition time = 5.12 min). Structural images were used for registration and to create lesion masks to optimize registration. rs-fMRI BOLD (blood oxygenation level-dependent) T2^*^-weighted series were obtained with a gradient echo-planar-imaging (EPI) acquisition (160 volumes, TR = 3.12 s, TE = 21 ms, flip angle = 90°, FOV = 288 mm, voxel size=3.0 × 3.0 × 3.0 mm, total acquisition time = 8.3 min). This voxel size was used to ensure an appropriate compromise between signal-to-noise ratio and spatial accuracy. During resting-state series acquisition, patients were instructed to lie still with their eyes open. Diffusion-weighted imaging, fluid-attenuated inversion recovery and fast low-angle shot sequences were also obtained but are not used in the current study. Total scanning time was 45 min.

For participants with an identified lesion, axial T1 images were used to draw a region of interest mask around the primary infarct using fslView (24). Masks were drawn by a trained neuroimaging researcher (MV), quality checked and modified as necessary by a senior neurologist (MP) to ensure they accurately represented the infarct. These masks were used during fMRIprep pre-processing for spatial normalization with Advanced Normalization Tools as a loss function. Lesion masks were also used in a second pipeline based on fMRIprep using methods suggested by Siegel and colleagues (25).

### Functional Data Processing and Stroke-Specific Processes

fMRIprep (version 1.2.5) was used to preprocess the anatomical and functional data (26) using a Nipype-based tool (27). Following pre-processing, manual quality assurance assessment was undertaken. Lesion masks were resampled from MNI anatomical resolution (1 mm iso) to the MNI functional resolution (3 mm iso).

Each T1w (T1-weighted) volume was corrected for INU (intensity non-uniformity) using N4BiasFieldCorrection v2.1.0 (28) and skull-stripped using antsBrainExtraction v2.1.0 (using the OASIS template). Brain surfaces were reconstructed using recon-all from FreeSurfer v6.0.1 (29), and the brain mask estimated previously was refined with a custom variation of the method to reconcile ANTs-derived and FreeSurfer-derived segmentation of the cortical gray-matter of Mindboggle (30). Spatial normalization to the ICBM 152 Non-linear Asymmetrical template version 2009c (31) was performed through non-linear registration with the antsRegistration tool of ANTs v2.1.0 (32), using brain-extracted versions of both T1w volume and template. Brain tissue segmentation of cerebrospinal fluid (CSF), white matter (WM) and gray-matter (GM) were performed on the brain-extracted T1w using fast (33) (FSL v5.0.9).

Functional data were slice time corrected using 3dShift from AFNI v16.2.07 (34) and motion-corrected using mcflirt [FSL v5.0.9 (24)]. “Fieldmap-less” distortion correction was performed by co-registering the functional image to the same-subject T1w image with intensity inverted (35) constrained with an average fieldmap template (36), implemented with antsRegistration (ANTs). This was followed by co-registration to the corresponding T1w using boundary-based registration (37) with 9 degrees of freedom, using bbregister (FreeSurfer v6.0.1). Motion correcting transformations, field distortion correcting warp, BOLD-to-T1w transformation and T1w-to-template (MNI) warp were concatenated and applied in a single step using antsApplyTransforms (ANTs v2.1.0) using Lanczos interpolation.

Frame-wise displacement (38) was calculated for each functional run using the implementation of Nipype. ICA-based Automatic Removal Of Motion Artifacts (AROMA) was used to generate aggressive noise regressors as well as to create a variant of data that is non-aggressively denoised (39). Many internal operations of FMRIPREP use Nilearn (40), principally within the BOLD-processing workflow. For more details of the pipeline see https://fmriprep.readthedocs.io/en/latest/workflows.html.

### Exclusions, Cleaning, and Smoothing

Participants with Framewise Displacement (41) >0.5 mm, visually incomplete separation of MELODIC motion components from known resting-state networks or artifact identified on the carpet plots (including high-frequency noise and motion-related spikes) were removed from further analysis. Unsmoothed, unfiltered and MNI normalized fMRI volumes were entered into a second pipeline for cleaning and smoothing. The pipeline was programmed using Python and made use of Numpy (42), Scipy, Pandas (43), Nipype (27) and Deepdish (https://github.com/uchicago-cs/deepdish). Motion and physiologically related artifact were regressed from the functional time series using Ordinary Least Squares Regression. The design matrix for all participants consisted of the time series from motion-related components identified by AROMA, CSF and a discrete high pass cosine filter (0.007 Hz/128 s).

### Lesion Masks

Residual images were z-scored, smoothed with a 6 mm full width half-maximum Gaussian kernel and parcellated with the MDSL atlas (44) using Nilearn (40). Lesions masks were used to attenuate the effects of the lesion related artifact on the ICA-smoothed BOLD signal using the method outlined by Yourganov et al. (45). Briefly, the overlap between the lesion mask and z score thresholded (*p* < 0.05) MELODIC component spatial maps was assessed using the Jaccard index (Scipy). If the Jaccard index was equal to or >5%, the component was considered to be significantly overlapping with the lesion mask to contribute mostly aberrant signal and added to the design matrix. If the component was already identified by AROMA as motion-related, it was not re-added.

**Figure 1:**
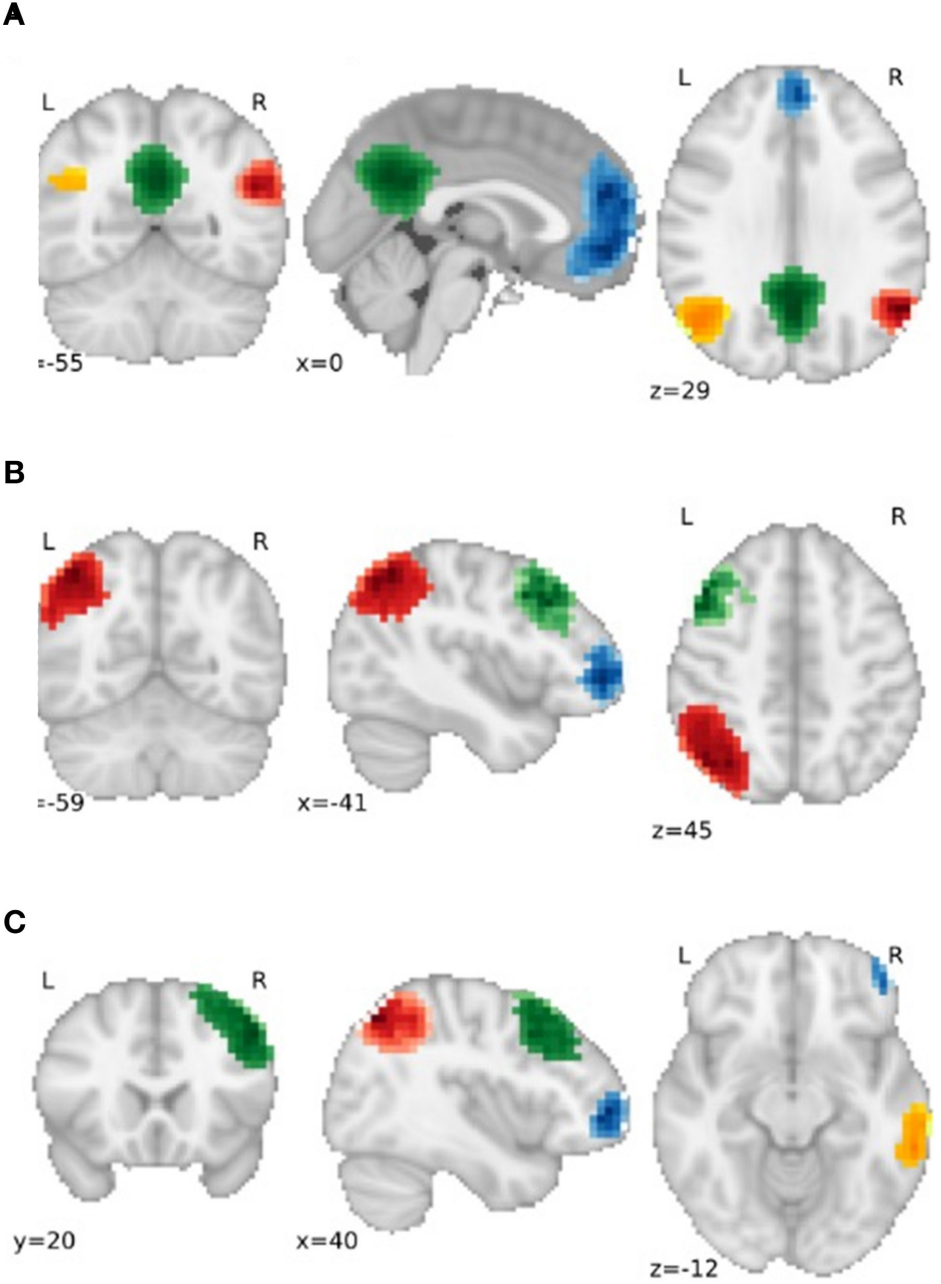
Gray matter regions used as nodes for the **(A)** default mode network (DMN), **(B)** Left Frontoparietal Network (FPN) and **(C)** Right FPN. For **(A)**, Blue = Frontal DMN, Green = Medial DMN, Orange = Left DMN, Red = Right DMN. For **(B)** and **(C)**, Blue = Dorsolateral Prefrontal node, Red = Parietal node, Green = Frontal Pole node, Orange = Posterior Temporal node.

Partial correlation was used to examine the functional connectivity between parcellations. As hypotheses referred to specific networks, we only examined parcellations associated with the left and right frontoparietal and the default mode networks (see Figure 1 for information regarding the spatial distribution and number of extracted nodes for each network). The differences between functional connections between groups were investigated with and without the addition of confounders age and vascular risk factors (i.e., the corrected and raw models, respectively).

### Cognitive Measures

#### Montreal Cognitive Assessment (MoCA)

The MoCA assesses short-term memory, visuospatial skills, executive functions, attention, language, and orientation. Scores below 23 indicate mild cognitive impairment and below 18 suggest dementia (46, 47).

#### Trail Making Test (TMT)

The TMT measures visual attention and task switching and is a validated indicator of brain damage (48). In part A, participants are instructed to connect numbers in ascending order from 1 to 25 and in Part B, in an ascending pattern, alternating between numbers and letters (i.e., 1-A-2-B, etc.). The TMT is scored by subtracting the total time taken for Part A from total time for Part B with higher scores indicating poorer performance.

#### National Institute of Health (NIH) Toolbox

Six cognitive tests from the NIH Toolbox cognition battery measuring crystallized intelligence, processing speed, memory, and executive functions were administered using the application on an iPad following standardized procedures as per the administration manual (49, 50). The NIH Toolbox shows acceptable internal consistency, test-retest reliability and validity (51, 52).

##### List Sorting Working Memory Task

List sorting working memory task assesses working memory. Participants recall and sequence a list of foods and animals presented orally and visually from smallest to largest. The first condition includes only food or animals and the second condition involves both food and animals, where foods are listed first from smallest to largest followed by animals. Scores were calculated using item response theory (IRT; a score derived from each participant's overall ability).

##### Pattern Comparison Processing Speed Task

Pattern comparison processing speed task measures processing speed by using IRT and raw performance values [average reaction time (RT) and accuracy] to score participants ability to decide whether two images presented side by side are the same or not.

##### Auditory Verbal Learning Task

Auditory verbal learning task measures short-term episodic memory. Participants are asked to verbally recall as many words as possible from a list of 15 unrelated words. The list was presented orally three times and the total score was determined by each correctly remembered word. Scores were determined by the number of total words recalled.

##### Dimensional Change Card Sort Task

Dimensional change card sort task assesses executive functions by asking participants to match bivalent stimuli to a target picture with two dimensions (shape and color) according to a word cue (shape or color). This requires participants to either switch or repeat the task (e.g., shape, shape, color—where the color trial is a switch trial). A computed score was created based on a combination of accuracy and RT performance. Raw performance values (average accuracy and RT) were also obtained.

##### Oral Reading Recognition Task

Examines reading decoding skills. Participants pronounce words displayed on an iPad with the test administrator scoring correct or incorrect. Scores were calculated using IRT.

##### Picture Vocabulary Task

Measures receptive vocabulary. After hearing a word, participants select one of four pictures that most closely matches the meaning of the word. Scores were calculated using IRT.

##### Crystallized Ability Composite Score

It was computed by averaging the Oral Reading Recognition and Picture Vocabulary Tests.

All scores, excluding the Auditory Verbal Learning Test, were corrected against the entire NIH Toolbox national US registry with a normative mean of 100 and a standard deviation of 15. A score of 85 and 115 indicates performance one standard deviation below and above the mean, respectively. Raw scores from the auditory verbal learning task were uncorrected, however, NIH normative data indicated a mean of 19.04 (SD = 9.08) for participants 60–69 years and a mean of 15.14 (SD = 6.14) for people 70–85 years.

### Statistical Analysis

SPSS (version 25.0) was used to examine the main effects of group (CVE vs. control and minor stroke vs. TIA) on cognitive performance. Differences were examined between the control and the cerebrovascular Event (CVE) groups and between CVE subgroups with minor stroke vs. TIA. Continuous variables were investigated using a one-way ANOVA and categorical variables using a chi-square test. An ANCOVA was used to investigate the effect of group on each cognitive measure while controlling for the hypothesized confounders: age and number of vascular risk factors. These confounders were chosen based on Munir et al. (11) and Nicolas et al. (6). As the current study aims to investigate the independent effect of CVE on cognitive ability, adjusting for age and the presence of vascular risk factors appeared appropriate.

Permutation analysis was used to investigate the significant group differences in rsFC between nodes (results shown in **Table 4**). Specifically, a null distribution was created using 10,000 permutations for each of the statistical connections (i.e., correlations between two parcellations) between nodes and compared to the observed *t*-value. There were a total of 11 nodes examined of which there were 55 pairwise combinations. MaxT correction was used for multiple comparisons (53), which is robust to outliers. Then, to investigate these group differences after controlling for the effects of age and vascular risk factors, these variables were regressed from each cognitive score using the Huber Regression (a method robust to the effects of outliers) in sci-kit learn (54) (epsilon =1.35, alpha = 1e-3, tol = 1e-5).

Significant associations in rsFC between nodes and cognitive performance and group interaction effects between rsFC and cognitive performance were determined using Manley's method {i.e., generating the null distribution using unresricted sampling. [See (55)]} and permutation tests (10,000 permutations were used to calculate a null distribution for each parameter). A Huber Regression was used to investigate the linear association between cognitive performance and functional connectivity, and the differences in these associations between groups. Then, to investigate these interactions without the effects of age and vascular risk factors, the effects of these variables were regressed from each cognitive and rsFC score. The cognitive and rsFC residuals without the effects of age and vascular risk factors are shown in **Figure 4**. As we focus on interaction effects between rsFC, group and cognitive performance, no multiple comparison correction was used for the regression analysis.

## Results

In total, 123 participants were recruited, including 15 patients from a neurology clinic, 82 patients from the INSIST study and 26 healthy controls.

Participants were excluded from analyses if they did not complete the second testing session (19 participants), showed a large neural abnormality (i.e., cyst or tumor; 2 participants), did not complete the MRI (9 participants) or had severe motion artifacts in the MRI data (10 participants). Patients from the larger INSIST-COG [see (6)] study that did not have a clinically adjudicated vascular event (i.e., patients who were adjudicated as having had a mimic CVE including seizure, migraine or visual disturbance *N* = 21) were not included in the current analysis as the current study was interested only in the differences between minor CVE patients and controls. One control was excluded due to a neural abnormalities that may have affected the functional connectivity analysis. The final sample included 62 participants of whom 17 had a minor stroke as identified on MRI, 25 had a TIA and 20 were controls.

As shown in Table 3, compared to the Control group, the CVE group had significantly fewer years of formal education and greater prevalence of males, hypertension and vascular risk factors (all *p* < 0.029). Minor stroke patients also had a significantly greater prevalence of hypertension and vascular risk factors than TIA patients (both *p* < 0.038). Of the minor stroke patients, structural imaging showed that three patients (18%) had a right-hemisphere ischaemic lesion, six (36%) had a left-hemisphere lesion, four (24%) had a lesion in both hemispheres, one (6%) had a lesion in the pons, one (6%) had a lesion in the basal ganglia and two (12%) had multiple middle cerebral artery infarcts. As the sample was small, we could not examine the effects of lesion location.

**Table 3:** Group differences in demographics and cognitive ability.

	**Cerebrovascular event** ***N*** **=** **42 (68%)**		**Control*N* =20 (32%)**	**Cerebrovascular event vs. control**		**Minor stroke vs. TIA**	
	**Minor stroke *N* = 17 (40%)**	**TIA*N* = 25(37%)**		**(df) F/Chi**	***p***	**(df) F/Chi^**2**^**	***p***
Age (Mean ± SD)	71.6 ± 10.4	69.9 ± 8.2	65.8 ± 9.2	(1, 61) 3.72	0.059	(1, 41) 0.35	0.550
Sex (% male)	13 (77)	16 (64)	8 (40)	(2) 4.75	0.029	(1) 0.74	0.391
Years education (Mean ± SD)	11.9 ± 2.2	12.9 ± 3.5	14.4 ± 2.6	(1, 61) 5.84	0.019	(1, 41) 1.46	0.234
Years since event (Mean ± SD)	4.2 ± 3.5	3.6 ± 1.1				(1, 41) 0.83	0.367
Montreal cognitive assessment	25.67 ± 2.5	26.7 ± 2.4	27.4 ± 1.8	(1, 59) 2.99	0.089	(1, 40) 2.37	0.132
**Vascular risk factors, n (%)**							
Hypertension	13 (77)	11 (44)	5 (25)	(1) 5.63	0.018	(1) 4.36	0.037
Hyperlipidaemia	8 (47)	10 (40)	4 (20)	(1) 3.09	0.079	(1) 0.21	0.650
Myocardial infarct	2 (12)	0	0	(1) 0.98	0.321	(1) 3.09	0.079
Angina	1 (6)	0	0	(1) 0.49	0.487	(1) 1.51	0.220
Peripheral vascular disease	1 (6)	0	0	(1) 0.48	0.487	(1) 1.51	0.220
Atrial fibrillation	2 (12)	2 (8)	3 (15)	(1) 0.41	0.524	(1) 1.67	0.683
Diabetes	3 (18)	2 (8)	0	(1) 2.59	0.108	(1) 0.90	0.343
Number of VRF (Mean ± SD)	1.8 ± 1.3	1.0 ± 1.0	0.6 ± 0.7	(1, 61) 6.22	0.015	(1, 41) 4.64	0.038
**Cognitive measures (Mean** **±** **SD)**							
Trail making task
*TMTa*	36.1 ± 13.2	32.2 ± 14.2	27.6 ± 4.9	(1, 60) 4.45	0.039	(1, 40) 1.09	0.303
*TMTb*	114.9 ± 89.7	84.12± 66.1	57.9 ± 17.9	(1, 59) 5.01	0.029	(1, 39) 1.55	0.220
*TMT B-A*	79.5 ± 81.1	51.7 ± 53.9	30.3 ± 16.3	(1, 59) 4.62	0.036	(1, 39) 1.56	0.219
List sorting working memory	93.9 ± 9.9	97.6 ± 11.2	100.2 ± 8.2	(1, 59) 2.65	0.109	(1, 39) 1.11	0.299
Pattern comparison processing speed							
*Composite score*	82.1 ± 15.0	81.3 ± 12.7	87.7 ± 14.4	(1, 58) 2.58	0.114	(1, 38) 0.03	0.858
*Accuracy %*	98.9 ± 1.9	98.9 ± 2.1	99.2 ± 1.5	(1, 59) 0.42	0.518	(1, 39) 0.00	0.986
*Reaction time*	2.6 ± 0.7	2.6 ± 0.6	2.4 ± 0.5	(1, 59) 2.39	0.128	(1, 39) 0.03	0.857
Auditory verbal learning	19.1 ± 4.9	21.2 ± 6.4	24.1 ± 4.1	(1, 58) 6.16	0.016	(1, 39) 1.27	0.266
Dimensional change card sort							
*Composite score*	94.9 ± 8.7	95.9 ± 11.5	103.1 ± 6.7	(1, 58) 9.54	0.003	(1, 38) 0.09	0.771
*Accuracy %*	92.1 ± 11.3	92.3 ± 11.8	96.6 ± 4.7	(1, 58) 2.87	0.096	(1, 38) 0.00	0.958
*Reaction time*	1.1 ± 0.4	1.0 ± 0.3	0.8 ± 0.2	(1, 58) 9.32	0.003	(1, 38) 0.70	0.408
Oral reading recognition	109.4 ± 6.9	111.1 ± 6.0	112.7 ± 5.8	(1, 59) 2.25	0.139	(1, 39) 0.60	0.443
Picture vocabulary	110.7 ± 8.4	114.4 ± 8.3	116.5 ± 8.7	(1, 59) 2.38	0.128	(1, 39) 1.91	0.175
Crystallized composite score	110.4 ± 7.3	113.1 ± 7.2	115.2 ± 7.5	(1, 58) 2.47	0.122	(1, 39) 1.38	0.248

*VRF, vascular risk factors; SD, standard deviation. Gray p-values on cognitive measures indicate those that remained significant after correcting for age and number of vascular risk factors*.

### Cognitive Measures

The CVE group performed significantly lower than the Control group on the Auditory Verbal Learning task, the composite and reaction time (RT) measures of the Dimensional Change Card Sort task, and all components of the Trail Making Task (all *p* < 0.039; Table 3). After controlling for age and number of vascular risk factors, group differences remained only for the composite and RT components of the Dimensional Change Card Sort task (*p* = 0.033 and *p* = 0.046, respectively; data not tabulated). Minor stroke and TIA patients did not differ on any cognitive tasks (all *p* > 0.132, Table 3).

### Resting-State Functional Connectivity Differences

**Figure 2:**
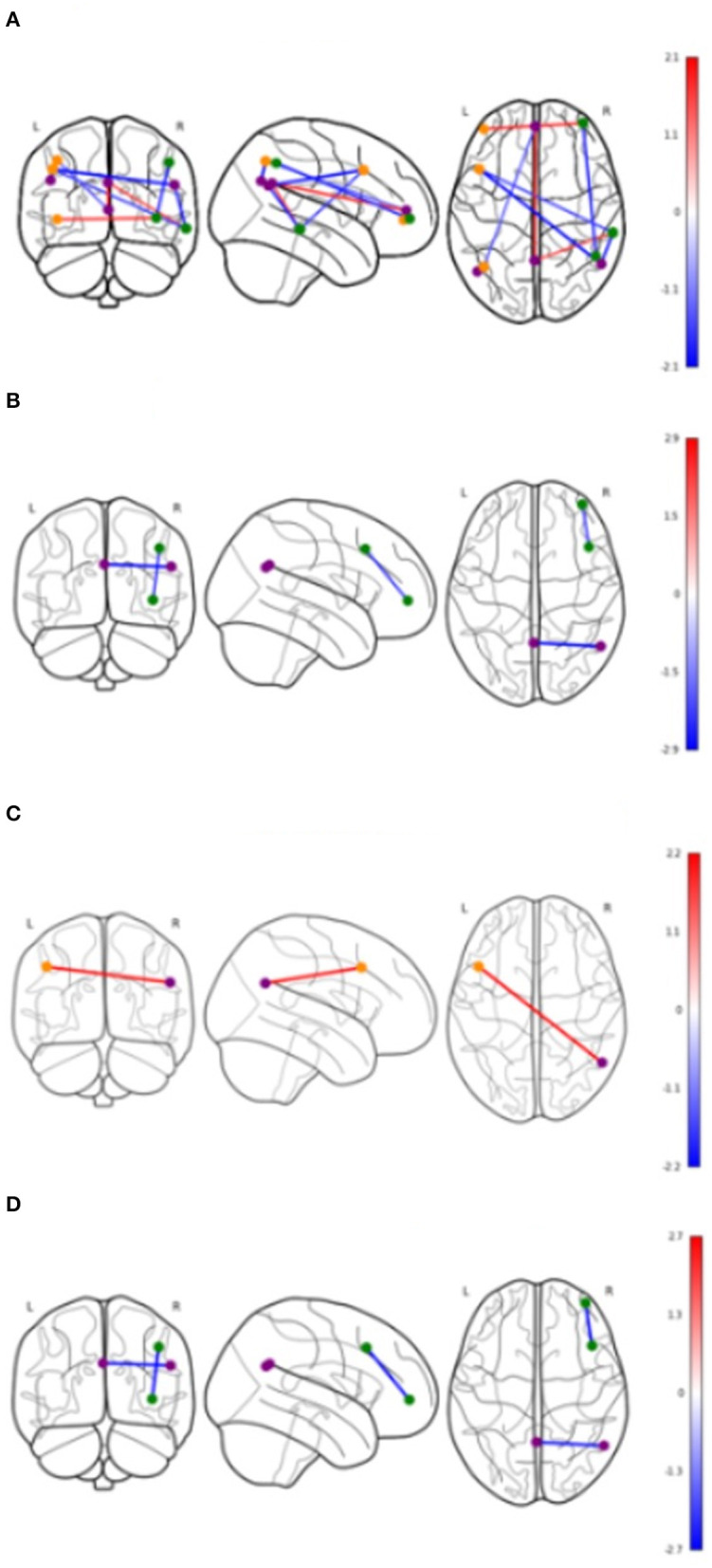
Significant differences in functional connectivity between nodes by groups. Values shown in color bars are *t*-values. Line color represents *t*-values (warm = positive, cool = negative). Nodes are colored based on the network they belong to. Purple = DMN, Orange = Left FPN, Green = Right FPN. **(A)** Cerebrovascular event vs. Control (uncorrected). **(B)** Minor stroke vs. TIA (uncorrected). **(C)** Cerebrovascular event vs. Control (corrected). **(D)** Minor stroke vs. TIA (corrected).

Table 4 shows significant group differences in the strength of rsFC between nodes extracted from the DMN and the left and right FPN network (see Figure 2 for a visual schematic of significant differences). Node location was then mapped to the corresponding network, e.g., connectivity between the left parietal FrP node (located in the FPN) and the left DMN node (located in the DMN) corresponds to connectivity between the FPN and the DMN.

**Table 4:** Significant groups differences in the strength of rsFC between nodes extracted from the DMN and the FPN network.

**Connectivity between nodes**	**Connectivity between corresponding network(s)**	**Observed *t*-value**	p^*^
**Raw model**			
**Control vs. cerebrovascular event**			
F DMN node ⇔ M DMN node	DMN ⇔ DMN	−1.81	0.011
R posterior temporal node ⇔ L dorsolateral prefrontal node	FPN ⇔ FPN	1.62	0.021
R frontal pole node ⇔ L frontal pole node	FPN ⇔ FPN	−1.85	0.008
R parietal node ⇔ R frontal pole node	FPN ⇔ FPN	1.85	0.009
R posterior temporal node ⇔ M DMN node	FPN ⇔DMN	−1.55	0.028
R posterior temporal node ⇔ R DMN node	FPN ⇔DMN	1.89	0.006
L parietal node ⇔ L DMN node	FPN ⇔DMN	2.04	0.003
L parietal node ⇔ F DMN node	FPN ⇔DMN	1.41	0.044
L dorsolateral prefrontal node ⇔R DMN node	FPN ⇔DMN	2.12	0.002
Minor stroke vs. TIA			
R DMN node ⇔ M DMN node	DMN ⇔ DMN	2.95	0.006
R frontal pole node ⇔ R dorsolateral prefrontal node	FPN ⇔ FPN	2.35	0.022
**Corrected model (age and vascular risk factors)**			
Control vs. cerebrovascular event			
L Dorsolateral prefrontal node ⇔R DMN node	FPN ⇔DMN	−2.18	0.035
Minor stroke vs. TIA			
R DMN node ⇔ M DMN node	DMN ⇔ DMN	2.63	0.013
R frontal pole node ⇔ R dorsolateral prefrontal node	FPN ⇔ FPN	2.68	0.013

* *maxT corrected*.

*Positive t observation indicates higher rsFC in left group compared to right group*.

*DMN, default mode network; FPN, frontoparietal network; L, left; R, right; F, front; M, medial*.

#### Control vs. Cerebrovascular Event Group

In Table 4, the raw model (top) refers to results before correcting for the effects of age and vascular risk factors, whereas the corrected model (bottom) refers to results that remained significant after correction. In the raw model, control and CVE groups differed in rsFC strength between nodes within DMN and FPN networks (all *p* < 0.021), as well as connectivity between nodes belonging to DMN and FPN networks (all *p* < 0.044). Compared to the CVE group, controls showed greater and reduced connectivity between nodes within the FPN, reduced connectivity between nodes within the DMN and both greater and reduced connectivity between nodes in the two different networks.

After controlling for age and number of vascular risk factors (corrected model), the only significant group difference was that rsFC between the left dorsolateral prefrontal node (FPN) and the right DMN node was weaker in controls than the CVE group (*p* = 0.035).

#### Minor Stroke vs. TIA

The minor stroke group showed significantly stronger rsFC between nodes within both DMN and FPN than the TIA group (*p* = 0.006 and *p* = 0.022, respectively), which remained significant after controlling for confounders (both *p* = 0.013).

### Interactions Between Cognition, Resting-State Functional Connectivity, and Group

**Figure 3:**
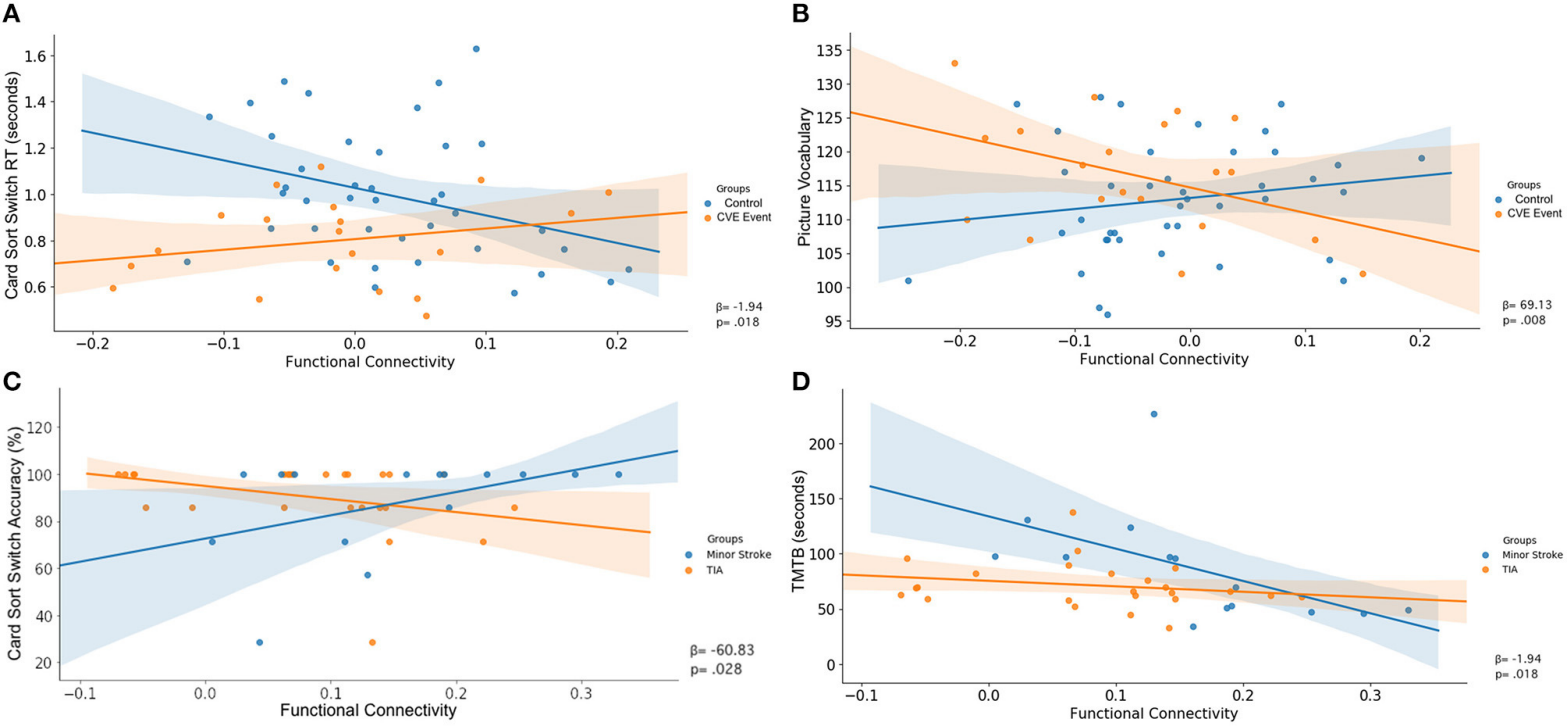
Uncorrected interaction of functional connectivity between two nodes (X-axis), cognitive performance (Y-axis) and group (legend). Shaded areas indicated the 95%-confidence intervals around the correlation coefficients. **(A)** FPN ⇔ FPN, **(B)** FPN ⇔ DMN, **(C)** FPN ⇔ FPN, **(D)** FPN ⇔ FPN.

**Figure 4:**
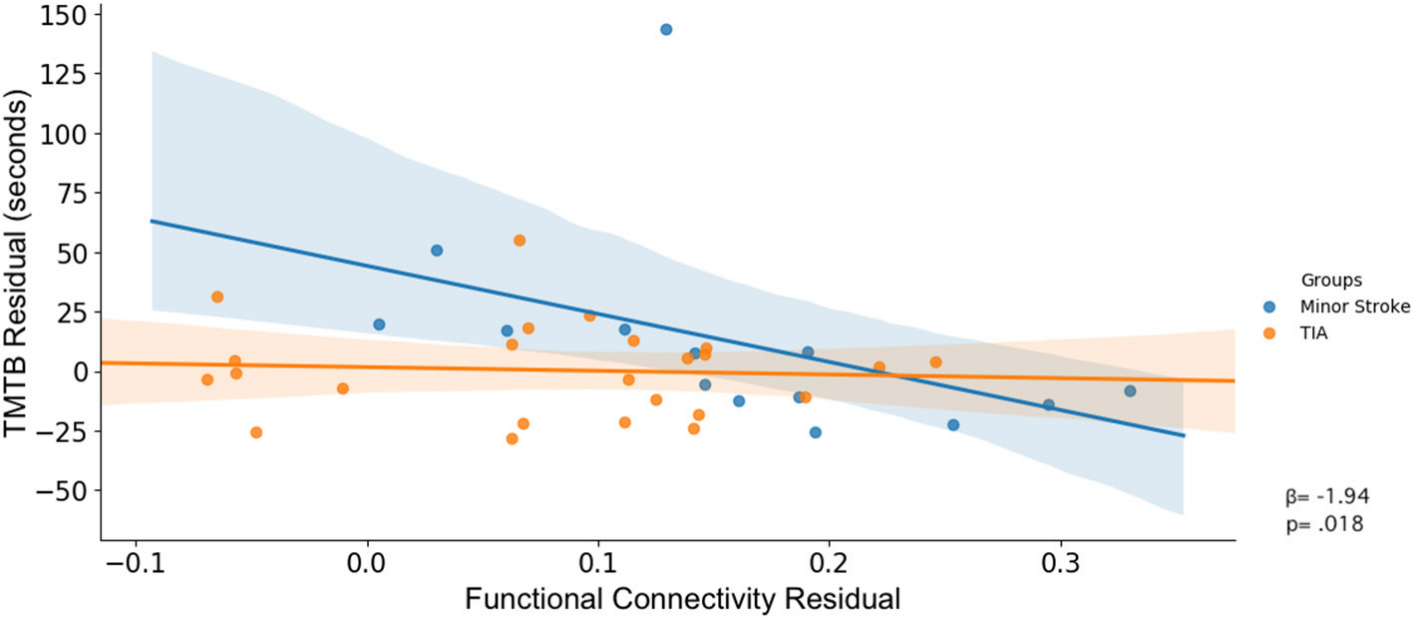
Corrected interaction of rsFC between FPN and FPN, TMT-B performance and group.

Table 5 shows significant associations between cognitive performance and rsFC, and the differences in these associations between groups. Figures 3, 4 display the interaction of rsFC between two nodes, cognitive performance and group.

**Table 5:** Interaction between functional connectivity and groups on cognitive outcomes.

	**Connectivity between (⇔) nodes**	**Connectivity between (⇔) network(s)**	**β**	***p***	**95% CI**
**Raw model**					
*Control vs. Cerebrovascular event*					
Dimensional change card sort					
*Switch trial RT*	R posterior temporal node ⇔ L dorsolateral prefrontal node	FPN ⇔ FPN	−1.94	0.018	−2.96 to −0.26
Picture Vocabulary	L parietal node ⇔ F DMN node	FPN ⇔DMN	69.13	0.008	−7.71 to 111.80
*Minor stroke vs. TIA*					
Dimensional change card sort					
*Switch trial accuracy*	R frontal pole node ⇔ R dorsolateral prefrontal node	FPN ⇔ FPN	−60.83	0.028	−159.28 to −18.94
Trail making task					
*TMTb*	R frontal pole node ⇔ R dorsolateral prefrontal node	FPN ⇔ FPN	216.8	0.030	98.07–411.74
**Corrected model (age and vascular risk)**					
*Minor stroke vs. TIA*					
Trail making task					
*TMTb*	R frontal pole node ⇔ R dorsolateral prefrontal node	FPN ⇔ FPN	157.82	0.047	45.21–329.49

*β, unstandardized coefficients. Positive β value indicates higher rsFC in the left group compared to the right group*.

#### Control vs. Cerebrovascular Event

There was an interaction between group, RT on the switch trials of the Dimensional Change Card Sort test and connectivity across nodes within the FPN (β = −1.94, *p* = 0.018; Table 5). As shown in Figure 3A, greater rsFC in FPN was associated with faster switch trial RT for the control group, but this CVE group showed a weak effect in the opposite direction.

Further, there was an interaction between group, performance on the Picture Vocabulary test and rsFC between nodes comprising the FPN and DMN (β = 69.13, *p* = 0.008). As shown in Figure 3B, greater connectivity between FPN and DMN was associated with higher vocabulary scores in the control group, whereas CVE patients showed a strong trend in the opposite direction.

Both of these interactions were removed after controlling for potential confounders (both *p* > 0.050).

#### Minor Stroke vs. TIA

There was an interaction between group, accuracy on the switch trials of the Dimensional Change Card Sort test and connectivity across nodes within the FPN (β = −60.83, *p* = 0.028; Table 5). As shown in Figure 3C, increased rsFC within the FPN was associated with greater accuracy in minor stroke patients and with little effect on TIA patients.

There was an interaction between group, TMTb performance and connectivity across nodes within the FPN (β = 216.80, *p* = 0.030). As shown in Figure 3D, increased rsFC within the FPN was associated with faster completion time for minor stroke patients with little effect on TIA patients.

After controlling for confounders, only the latter interaction remained (β = 157.82, *p* = 0.047; Figure 4).

## Discussion

We examined whether there were persistent cognitive and resting-state functional connectivity differences between (a) healthy controls and cerebrovascular event patients and (b) between minor stroke and TIA patients approximately 4 years following their event. In line with our hypothesis, CVE patients had poorer performance on measures of executive functioning and greater inter-network rsFC between nodes connecting the FPN and DMN when compared to healthy controls. Interestingly, when compared to TIA patients, minor stroke patients with MRI-defined brain infarction displayed greater intra-network rsFC in the DMN and the FPN. Finally, the strength of this intra-network rsFC within the FPN was associated with differential performance in minor stroke and TIA patients on the B component of the TMT, a measure sensitive to executive functioning.

Executive functioning ability is associated with rsFC between task-positive (FPN) and task-negative (DMN) networks (56). Reduced connectivity between these networks is believed to demonstrate the ability to suppress task-negative networks and increase activation of task-positive networks during a cognitive task (57, 58). After controlling for confounders, controls displayed better performance on measures of executive functions and reduced rsFC between task-positive and task-negative networks, when compared to CVE patients. So controls appear to more successfully suppress DMN activity and activate FPN when completing executive function tasks. While the absence of this relationship between performance and rsFC in participants with a prior CVE is consistent with persistent neural changes, these findings need to be interpreted with caution, as we found no significant interaction between rsFC, cognitive performance and group. This is likely due to the relatively small and heterogeneous CVE group which included patients with and without a permanent infarct, as well as heterogenous lesion locations in the minor stroke group. Moreover, although the CVE group had more males than the control group, we did not control for this as it is consistent with the consistent with sex distribution in typical stroke populations and the small sample size limits the effectiveness of correction.

While there were no cognitive differences between CVE patients with and without an infarct (i.e., minor stroke and TIA, respectively), minor stroke patients displayed greater intranetwork rsFC within both the DMN (right to medial DMN regions) and the FPN (right frontal pole to right DLPFC). Each of these regions is associated with executive functions including monitoring outcomes and working memory (59–61). Previous studies have found the DMN is linked to internally oriented mentation (62) and the DLPFC and frontal pole have been demonstrated to be involved in information manipulation, controlling working memory and organizing and cataloging information (63, 64) which are all components of executive functions. While it is unknown why these neural regions were over-activated in minor stroke compared to TIA patients, it is well-known that both acute stroke (65, 66) and TIA (14, 15) can lead to abnormal rsFC. However, to the best of our knowledge, there are currently no studies investigating the differences between rsFC in minor stroke and TIA patients and thus, future research is required to further investigate these findings. Moreover, increased connectivity within the FPN was associated with better performance on the B component of the TMT task in minor stroke patients but appeared to have little effect in TIA patients. As minor stroke patients in the current study are characterized by the presence of a persistent infarction, we speculate that differences between groups may be attributed to the use of compensatory processes (e.g., recruiting additional frontal neural resources or functionally reorganizing neural regions) to overcome infarct-related damage which in turn resulted in them performing as well as TIA patients. This conclusion, however, requires further investigation using a larger cohort of patients with homogenous brain lesions and demographics to successfully generalize and interpret such results. Together, these findings provide evidence that the presence of ischaemia following a minor neurological event results in significant, long-term connectivity differences, the strength of which can differentially affect executive functioning.

There are potential limitations with deriving measures of rsFC in ischaemic patients. Although we used the current gold standards for quantifying stroke-affected BOLD (67) to reduce the effect of individual differences across patients (e.g., differing lesion/occlusion locations and the severity of neural damage), it is still possible that there was some signal artifact. Additionally, the parcellation scheme assumes that individuals have common functional parcellations in standard space, so individual differences due to different lesion/occlusion locations may result in measurement error (68). Further, functional reorganization can occur following an ischemic event (67) and this can shift functional boundaries and delay the BOLD response, resulting in individual rsFC estimate errors. We used an Independent Component Analysis method (45) to overcome this delay in minor stroke patients. However, such a method is unavailable for TIA patients who may also experience a delay. Finally, while BOLD is an indirect measure of neural activity, the precise functional significance of rsFC derived from the BOLD signal and its relationship to excitatory or inhibitory brain processes remains under investigation.

In conclusion, we found poorer executive functions and disrupted inter-network rsFC between the DMN and FPN in minor CVE patients ~4 years after the event. These findings indicate that the presence of a minor CVE is independently associated with persistent neurobiological and functional consequences. Further, while there were no cognitive differences between minor cerebrovascular patients with and without evidence of a neural infarct, these subgroups showed different patterns of rsFC within nodes of the FPN which differentially affected performance on a measure of executive functioning. Together, these findings provide evidence that patients with and without a neural infarct show different patterns of rsFC which can affect cognitive performance. This emphasizes the importance of event prevention and the need for the development of restorative approaches aimed at reducing the impact of minor stroke and TIA on long-term cognition and quality of life.

## Data Availability Statement

The raw data supporting the conclusions of this article will be made available by the authors, without undue reservation.

## Ethics Statement

The studies involving human participants were reviewed and approved by Hunter New England Human Research Ethics Committee (12/04/18/4.02). The patients/participants provided their written informed consent to participate in this study.

## Author Contributions

KN: investigation, writing—original draft, project administration, data curation, formal analysis, methodology, and conceptualization. PG: software, formal analysis, writing—review & editing, methodology, and visualization. MV: writing—review & editing and formal analysis. PM: supervision and writing—review & editing. AB: supervision, writing—review & editing, conceptualization, methodology, and funding acquisition. CL: supervision, writing—review & editing, and funding acquisition. MP: writing—review & editing. FK: supervision, writing—review & editing, and conceptualization. All authors contributed to the article and approved the submitted version.

## Conflict of Interest

The authors declare that the research was conducted in the absence of any commercial or financial relationships that could be construed as a potential conflict of interest.
